# Bilateral Renal Colic as an Initial Presentation of Erdheim-Chester Disease

**DOI:** 10.1155/2019/4670376

**Published:** 2019-12-29

**Authors:** Julien Sarkis, Fady Haddad, Sarah Nasr, Elie Hanna, Ahmad Mroueh, Elie Nemr

**Affiliations:** ^1^Department of Urology, Hôtel-Dieu de France, Beirut, Lebanon; ^2^Department of Internal Medicine, Hôtel-Dieu de France, Beirut, Lebanon; ^3^Department of Pathology, Hôtel-Dieu de France, Beirut, Lebanon; ^4^Department of Nephrology, Hôtel-Dieu de France, Beirut, Lebanon

## Abstract

Erdheim-Chester disease (ECD) is a rare non-Langerhans cells histiocytosis characterized by multiorgan involvement, with renal-ECD documented in over one-third of patients. Renal disease is generally asymptomatic, rarely causing hydronephrosis and kidney impairment. In addition, the diverse clinical picture of Erdheim-Chester disease arises slowly with sequential manifestations. We present a rare case of a 75-year-old woman on long-term treatment for panhypopituitarism and steroid therapy for vasculitis, presenting to the emergency department with bilateral renal colic and acute kidney injury. Abdominopelvic CT scan revealed renal infiltration with signs of retroperitoneal fibrosis and hydronephrosis. Kidney CT-guided biopsy and 18-fluorodeoxyglucose (FDG) positron emission tomography whole body scan as well as the history of hypopituitarism and vasculitis confirmed the diagnosis of Erdheim-Chester disease. Proper therapy with interferon-*α* was started. This case describes the multifaced manifestation of this disease and the difficulty to establish the diagnosis, as well as the pivotal role that a urologist can play in its management.

## 1. Background

Erdheim-Chester disease (ECD) is a rare non-Langerhans histiocytic disorder with fewer than 600 cases reported in the literature [1]. It is characterized by a multiorgan involvement with a special affinity to the skeletal and the central nervous systems [2]. Asymptomatic retroperitoneal involvement is seen in more than one-third of patients with ECD [3]. However renal involvement with consequent hydronephrosis and impairment was described in only 6% of the population [4]. This diverse clinical picture of ECD arises as a slowly forming mosaic with sequential manifestations, leading to frequent delay in diagnosis.

Here, we present a rare case of a 75-year-old woman on long-term treatment for panhypopituitarism and steroid therapy for vasculitis, diagnosed of ECD following bilateral renal colic.

## 2. Case Presentation

It is the case of a 75-year-old Caucasian woman with a medical history of central diabetes insipidus and central hypothyroidism, both diagnosed in 2005. Six years ago, she was diagnosed with large vessel vasculitis following symptoms of headache associated to elevated C-reactive protein (CRP) and aortic involvement on PET/CT scan. Steroid-based immunosuppressive therapy was started. Methotrexate was first given in adjunction to prednisone but later discontinued due to severe myelosuppression. Both temporal artery biopsies were negative for Horton's disease. Patient is hypertensive and obese with recurrent osteoporotic vertebral compression fractures requiring cementoplasty and a sedentary lifestyle due to lower limb pain limiting her daily activity.

She presented to our emergency department due to increasing bilateral flank pain that started several weeks ago, associated to recent episodes of vomiting and general weakness. On physical exam, patient was alert and conscious, afebrile with stable vital signs. Abdomen was soft without any point tenderness. Lumbar punch was positive bilaterally. Investigations revealed acute kidney injury with a creatinine of 308 *μ*mol/L (3.8 mg/dL), and bilateral hydronephrosis with renal pelvic thickening on imaging. The patient underwent urgent bilateral ureteral stent insertion, resulting in an improved renal function (creatinine of 65 *μ*mol/L or 0.73 mg/dL on day 5 after surgery).

**Figure 1:**
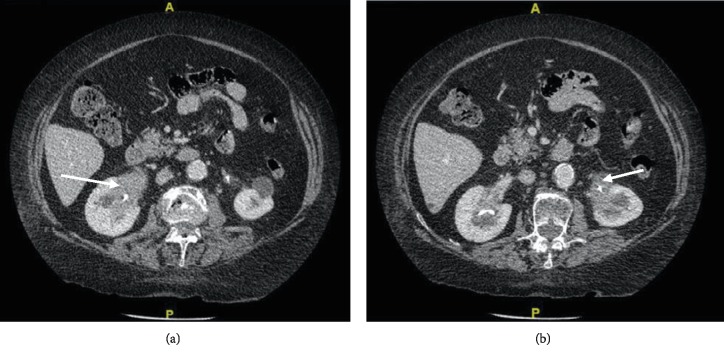
Injected computed tomography of the abdomen after bilateral ureteral stent insertion: *arrows* showing the infiltration of the right (a) and left (b) kidneys by a homogeneous tissue enclosing the pelvis, signs of retroperitoneal fibrosis. No characteristic “hairy kidney” appearance and no signs of aortic involvement.

Due to the unusual renal involvement on imaging, as well as the patient's medical history of panhypopituitarism and vasculitis, a diagnosis of histiocytosis was suggested. An injected thoraco-abdominopelvic CT scan showed infiltration of both the kidney sinus by a homogeneous tissue enclosing the pelvis and the proximal ureters as well as the vascular structures of the renal hilum suggestive of retroperitoneal fibrosis (Figure 1). On bone windows, iliac wings were seen containing poorly defined sclerotic plaques, associated to bone lysis and fracture on the right side.

**Figure 2:**
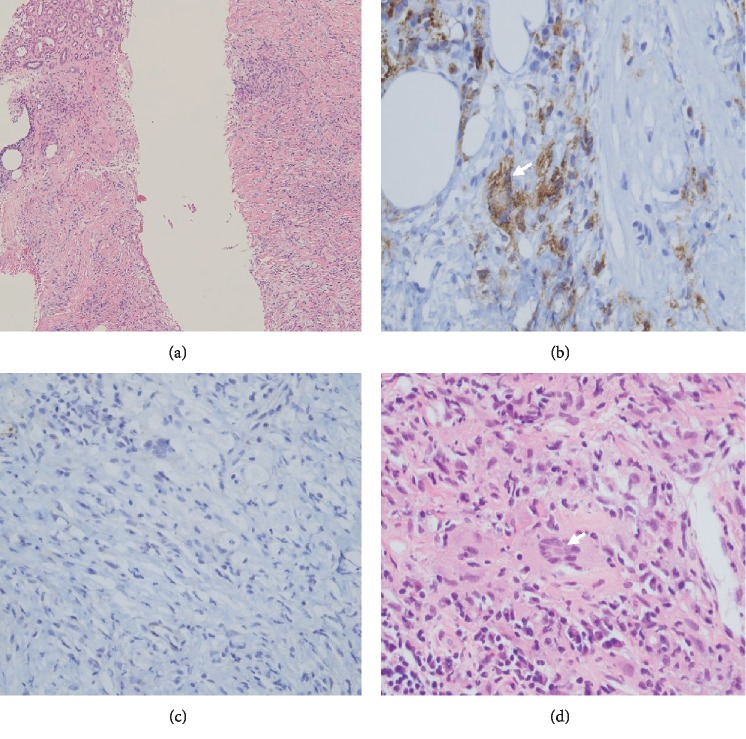
Histopathological finding of the renal specimen resected by CT-guided Tru-Cut biopsies: hematoxylin-eosin staining revealed a diffuse proliferation of histiocytes associated to lymphocytic and monocellular invasion, as well as important fibrotic involvement ((a) original magnification ×100). Immunochemistry demonstrated that histiocytes were strongly positive for CD68 (b) and negative for CD1a antigen (c) ((b, c) original magnification ×400). Touton-like multinucleated giant cells (arrowheads) are visible on both CD68 (b) and hematoxylin-eosin staining ((d) original magnification ×400).

In coordination with our interventional radiology team, multiple CT-guided Tru-Cut biopsies of the left thickened renal pelvis were obtained. The diagnosis of ECD was confirmed by pathology, with signs of retroperitoneal fibrosis, as well as histiocytosis associated to lymphocytic and monocellular invasion (Figure 2(a)). On immunohistochemical staining, histiocytes were CD68 positive (Figure 2(b)) but negative for CD1a antigen (Figure 2(c)). Multinucleated giant Touton cells were also visible (Figures 2(b) and 2(d)). IgG4 testing results on pathology and serology were both negative, ruling out IgG4-related retroperitoneal fibrosis.

**Figure 3:**
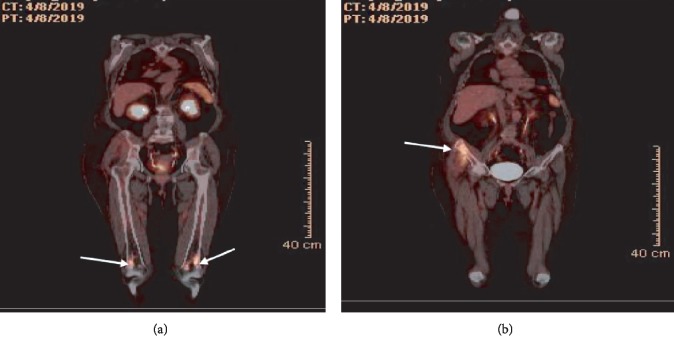
18-Fluorodeoxyglucose positron emission tomography (coronal views): ^18^FDG-PET scan showing FDG-avid sclerotic bone lesions in the distal femoral metadiaphysis bilaterally (a) as well as the right iliac bone involvement (b).

An 18-fluorodeoxyglucose (FDG) positron emission tomography whole body scan was realized to assess the extension of the disease revealed an intensely FDG-avid suprasellar soft tissue lesion, as well multiple FDG-avid sclerotic bone lesions in the distal femoral metadiaphysis bilaterally. An uptake of 18-FDG was also notable in the fractured right iliac bone (Figure 3). The diagnosis of ECD was confirmed, followed by negative BRAF mutation testing. The patient was discharged on interferon-*α* at day 17 after her admission, with a creatinine of 72 *μ*mol/L (0.81 mg/dL). An echocardiogram ruled out heart involvement. Her hospital course was complicated by a nonmassive pulmonary embolism with normal right ventricular function, treated by therapeutic anticoagulation.

## 3. Discussion and Conclusion

Histiocytosis is a group of rare diseases characterized by abnormal accumulation of macrophages, dendritic cells, or monocyte-derived cells in different tissues causing highly heterogenous clinical findings [5]. Erdheim-Chester disease (ECD) is an uncommon non-Langerhans cell histiocytosis distinguished on pathology by the presence of lipid-laden histiocytes that are immunochemically CD68 (histiocyte marker) positive but negative for CD1a and S100 (Langerhans cell markers). Multinucleated Touton giant cell's presence also favors the disease [6]. Like all histiocytosis, ECD has a multiorgan implication, but it is typically characterized by skeletal and central nervous system involvement. Skeletal lesions occur in 74% of patients with characteristic multifocal osteosclerotic lesions of the long bones [7]. The central nervous system is involved in half of the cases, with central diabetes insipidus being the most common manifestation of neuro-ECD [3]. Renally, a recent systematic review of the literature described asymptomatic retroperitoneal involvement in more than one third of patients [3]. However, renal involvement with consequent hydronephrosis and impairment was present in only 6% of the population, generally with the characteristic appearance of “hairy kidneys” on the CT scan [4].

The protean manifestations of ECD as well as its slow progression render its diagnosis challenging. Patients frequently face a delay in initiation of therapy often due to a difficulty in diagnosis. Our patient has suffered a delay of over 10 years since her initial diagnosis of central diabetes insipidus and hypothyroidism. Steroid and methotrexate started as treatment of her vascular involvement were the consequence of morbid drug side effects: corticosteroid-induced obesity and osteoporosis, as well as methotrexate-induced myelosuppression. Skeletal involvement was also present in our patient, leading to two episodes of vertebral fractures requiring cementoplasty as well as chronic lower limbs pain. However, bone disease was underlooked, and linked to chronic prednisone therapy. The presence of osteosclerosis of the iliac crests on CT as well as the history of vascular and central nervous disease, with the new image of signs of retroperitoneal fibrosis causing acute kidney injury finally pointed to ECD. Renal involvement in this case is atypical, consisting of symptomatic retroperitoneal fibrosis without the characteristic “hairy kidneys” on CT scan. Pathology is mandatory in confirming the diagnosis. In our case, CT-guided biopsies were of little morbidity and sufficient, showing the characteristic lipid-laden histiocytosis and monocellular invasion as well as signs of retroperitoneal fibrosis. The search of BRAF mutation was negative, rendering the use of BRAF-inhibitors impossible. Considered as first-line therapy in BRAF-negative patients, Interferon-*α* was started and response evaluation programmed.

This case typically describes the multifaced manifestation of ECD, as well as the difficulty to establish the diagnosis. Even though its clinical manifestations are well described in the literature, the diverse and multi-system involvement render the diagnosis challenging. Therefore, physicians should be familiar with this disease, sparing patients years of unnecessary treatments.
